# Genome-wide Fitness Profiles Reveal a Requirement for Autophagy During Yeast Fermentation

**DOI:** 10.1534/g3.111.000836

**Published:** 2011-10-01

**Authors:** Nina Piggott, Michael A. Cook, Mike Tyers, Vivien Measday

**Affiliations:** * Wine Research Centre, Faculty of Land and Food Systems, University of British Columbia, Vancouver, British Columbia, V6T 1Z4, Canada; † Centre for Systems Biology, Samuel Lunenfeld Research Institute, Mount Sinai Hospital, Toronto, Ontario, M5G 1X5, Canada; ‡ Department of Molecular and Medical Genetics, University of Toronto, Toronto, Ontario, M5S 1A8, Canada; § Wellcome Trust Centre for Cell Biology, School of Biological Sciences, University of Edinburgh, Edinburgh EH9 3JR, Scotland, United Kingdom

**Keywords:** *S. cerevisiae*, fermentation, fitness profiling, environmental stress, autophagy

## Abstract

The ability of cells to respond to environmental changes and adapt their metabolism enables cell survival under stressful conditions. The budding yeast *Saccharomyces cerevisiae (S. cerevisiae*) is particularly well adapted to the harsh conditions of anaerobic wine fermentation. However, *S. cerevisiae* gene function has not been previously systematically interrogated under conditions of industrial fermentation. We performed a genome-wide study of essential and nonessential *S. cerevisiae* gene requirements during grape juice fermentation to identify deletion strains that are either depleted or enriched within the viable fermentative population. Genes that function in autophagy and ubiquitin-proteasome degradation are required for optimal survival during fermentation, whereas genes that function in ribosome assembly and peroxisome biogenesis impair fitness during fermentation. We also uncover fermentation phenotypes for 139 uncharacterized genes with no previously known cellular function. We demonstrate that autophagy is induced early in wine fermentation in a nitrogen-replete environment, suggesting that autophagy may be triggered by other forms of stress that arise during fermentation. These results provide insights into the complex fermentation process and suggest possible means for improvement of industrial fermentation strains.

The budding yeast *S. cerevisiae* is a hallmark model organism for understanding cellular and molecular processes; it is also one of the most important industrial microorganisms for food and enzyme production. The ability of *S. cerevisiae* to rapidly adapt to changing environmental conditions, including survival in both aerobic and anaerobic conditions, and to out-compete other microbes by virtue of its high tolerance for ethanol has underpinned the propagation of *S. cerevisiae* strains optimized for fermentation performance. Evolutionary pressures acting on the *S. cerevisiae* genome have resulted in gains of genes to enable adaptation to anaerobic fermentation (Gordon *et al.* 2009). Although industrial fermentation is an anthropic environment, *S. cerevisiae* naturally proliferates in the interior of rotting fruit such as damaged grape berries, where it effectively creates a fermentative environment (Mortimer and Polsinelli 1999). In fact, the progenitor of the laboratory yeast strain S288C, which was the first eukaryotic genome to be sequenced, was isolated from a rotting fig in California in 1938 (Goffeau *et al.* 1996; Mortimer and Johnston 1986). Despite the explosion in genomics, proteomics, and systems biology since the sequencing of S288C, ∼1000 of the ∼6200 annotated yeast genes still have no known function (Pena-Castillo and Hughes 2007). However, to date, most high-throughput functional genomic analyses have been acquired under laboratory conditions that do not closely resemble the natural fermentative lifestyle of *S. cerevisiae*.

During fermentation of grape juice, *S. cerevisiae* is exposed to many stresses, including high osmolarity (20–40% equimolar glucose:fructose), organic acid stress (pH 3–3.5), limiting nitrogen, anaerobiosis, and ethanol toxicity [final concentration 12–15% (v/v)]. Whole-genome gene expression analysis of wine yeast strains during fermentation under wine-making conditions has demonstrated dramatic expression changes in ∼40% of the genome, including upregulation of stress response, energy production, and surprisingly, glucose repressed genes (Backhus *et al.* 2001; Marks *et al.* 2008; Perez-Ortin *et al.* 2002; Rossignol *et al.* 2003; Varela *et al.* 2005). Studies of short-term stress response in laboratory yeast strains have identified a signature environmental stress response of 10–20% of the genome to changes in temperature, nutrients, osmotic shock, and nutrient depletion (Causton *et al.* 2001; Gasch *et al.* 2000; Gasch and Werner-Washburne 2002). Although genome-wide expression data have provided valuable insights, gene mRNA expression profiles often do not correlate with gene requirement under specific conditions (Giaever *et al.* 2002; Winzeler *et al.* 1999). In addition, protein levels and function are often affected by post-translational modification in the absence of changes in gene expression. A comparison of the transcriptome and proteome of a wine yeast strain during fermentation revealed only a weak correlation between changes in mRNA and protein abundance at stationary phase (Rossignol *et al.* 2009). Thus, to gain insight as to how yeast cells sense and respond to environmental conditions, the functional requirement for each gene must be analyzed.

Laboratory strains of *S. cerevisiae* exhibit suboptimal fermentation performance compared with industrial *S. cerevisiae* strains because of their inability to convert all sugars present in grape must to ethanol (Pizarro *et al.* 2007). However, an auxotrophic laboratory strain of S288C is able to ferment grape juice to completion by supplementation of required amino acids and reduction of sugars (Harsch *et al.* 2009). Although earlier studies demonstrated aneuploidy in some wine yeast strains, recent karyotypic analysis of four commercial *S. cerevisiae* wine yeast strains revealed only small to moderate variations in gene copy number compared with S288C, with no major chromosomal rearrangements or abnormal chromosome numbers (Dunn *et al.* 2005; Pretorius 2000). In addition, genetic analysis of 45 commercial yeast strains showed that 40 strains were diploid whereas only five were aneuploid (Bradbury *et al.* 2006). Recent sequencing studies have provided the yeast community with new insight into the genomic variation between *S. cerevisiae* laboratory, industrial, clinical, and wild strains (Borneman *et al.* 2008; Liti *et al.* 2009; Novo *et al.* 2009; Schacherer *et al.* 2009; Wei *et al.* 2007). Genome sequencing and single nucleotide polymorphism analysis of 86 *S. cerevisiae* strains demonstrated that wine yeast strains from geographically distinct locations are closely related, suggesting a single domestication event (Liti *et al.* 2009; Schacherer *et al.* 2009). The genome of the commercial wine yeast strain EC1118 possesses three unique chromosomal regions encompassing 34 genes, of which two regions contain DNA from a non-*Saccharomyces* origin (Novo *et al.* 2009). Although these unique chromosomal regions are likely involved in the adaptation of EC1118 to industrial fermentation conditions, 99% of predicted EC1118 open reading frames (ORF) are in common with S288C (Novo *et al.* 2009). Likewise when the genomes of the wine yeast strain AWRI1631 and S288C were compared, although ∼68,000 single nucleotide variations were identified, the proteomes exhibited over 99.3% amino acid identity (Borneman *et al.* 2008). The fact that gene order, predicted ORFs, and proteomes are highly similar between *S. cerevisiae* S288C and wine yeast strains suggests that genomic technologies developed in S288C may be exploited to reveal gene functions required to cope with the dynamic stresses imposed by fermentation.

The comprehensive yeast gene deletion strain collection has enabled high-throughput screens for phenotypic traits of yeast genes; however, many of the physiological conditions tested are not relevant to industrial or natural yeast environments (Scherens and Goffeau 2004). A competitive fitness study of the heterozygous deletion mutant collection under conditions of nitrogen limitation revealed that impaired 26S proteasome function afforded a growth advantage, suggesting that a defect in protein degradation may be beneficial when nutrients are limiting (Delneri *et al.* 2008). Although the majority of the yeast genome is nonessential when grown in laboratory conditions, a comprehensive set of chemical genomic profiles uncovered a phenotype for 97% of yeast genes under chemical or environmental stress conditions (Hillenmeyer *et al.* 2008). Although a number of studies have assessed the deletion collection for ethanol sensitivity, very few genes appear in common across the different datasets, suggesting that the cellular response to ethanol may depend heavily upon precise experimental conditions (Fujita *et al.* 2006; Kubota *et al.* 2004; Kumar *et al.* 2008; Teixeira *et al.* 2009; Van Voorst *et al.* 2006; Yazawa *et al.* 2007; Yoshikawa *et al.* 2009). Finally, the deletion collection has been screened to identify yeast genes that confer resistance to inhibitors of bioethanol fermentation (Endo *et al.* 2008; Gorsich *et al.* 2006). Notably, genome-wide analysis of genetic requirements during wine fermentation has not been previously reported.

Here, we profile the genome-wide yeast deletion collection in S288C over a 14 day fermentation period to identify deletion strains that confer either a fitness defect or an advantage. Strains with fitness defects during fermentation were enriched for gene deletions in three major categories: autophagy, modification by ubiquitination, and proteasome degradation. Strains with a fitness advantage during fermentation were compromised for ribosomal proteins, peroxisome biogenesis, or phosphate homeostasis. Autophagy is the process whereby cytoplasmic components and excess organelles are degraded; we demonstrate that autophagy is induced under wine fermentation conditions. Finally, deletion of 139 uncharacterized genes alters yeast fitness during fermentation, suggesting that interrogation under nonstandard laboratory conditions will be required to uncover the function of many strongly selected genes in yeast evolution.

## Materials and Methods

### Yeast fermentation

The S288C diploid homozygous and heterozygous deletion pools were created as described (Ooi *et al.* 2001). The homozygous deletion pool (MAT a/α *gene XΔKanMX4/gene XΔKanMX4 his3Δ1/his3Δ1 leu2Δ0/leu2Δ0 ura3Δ0/ura3Δ0 met15Δ0/MET15lys2Δ0/LYS2*) was purchased from Invitrogen and contains 4653 homozygous deletions represented equally at a concentration of 2 × 10^7^ cells/mL. The heterozygous deletion pool (MAT a/α *gene XΔKanMX4/GENE X his3Δ1/his3Δ1 leu2Δ0/leu2Δ0 ura3Δ0/ura3Δ0 met15Δ0/MET15lys2Δ0/LYS2*) was generated in the following manner. Individual heterozygous deletion mutants (Open Biosystems, #YSC1055, 5797 unique ORFs) were pinned onto YPD plates (96 colonies per plate) containing G418 (200 μg/mL, Gibco #1181-031) and incubated at 25° for three days. Five mL of YPD (supplemented with 100 μg/mL G418) were added to each plate, cells were resuspended using a glass rod, and then transferred into a flask on ice. Glycerol was added to 15% final concentration, and cells were frozen in 1 mL aliquots (OD_600_ = 15.0). One biological replicate of the homozygous and heterozygous deletion pools (6 × 10^6^ cells), the S288C parental strain (BY4743), and the EC1118 strain were each grown overnight in rich glucose medium [YPD: 2% glucose (Fisher Scientific #D15-12), 2% bacto-peptone (BD #DF0118072), 1% yeast extract (BD #DF0127071)] to mid–log phase, washed in sterile water, and then resuspended in filter-sterilized synthetic grape juice [10% glucose, 10% fructose (Acros Organics #161350025), 0.45% malic acid (Sigma-Aldrich #M1000), 0.45% tartaric acid (Sigma-Aldrich #T1807), 0.03% citric acid (Sigma-Aldrich #C83155), 0.2% (NH_4_)_2_SO_4_ (Fisher #A702-3), 0.17% yeast nitrogen base without amino acids and ammonium sulfate (Difco #DF0335159), 0.1% (v/v) Tween 80 (Sigma P4780), 0.8 mM tryptophan (Sigma #T0254), 0.2 mM uracil (Sigma #U0750), 0.16 mM adenine (Sigma #A9126), 0.2 mM lysine (Sigma #L5501), 2 mM leucine (Sigma #L8000), 0.2 mM histidine (Sigma #H8125), adjusted to pH 3.0)] and inoculated into 500 mL of filter-sterilized synthetic grape juice at 2 × 10^6^ cells/mL in a 500 mL Kimax bottle fitted with a vapor lock. Fermentations were carried out at 21°. The first time point was taken 24 h post inoculation into synthetic grape juice and was used as a control in all hybridizations. Subsequent time points were taken at days 2, 4, 6, 8, 10, and 14 by withdrawal of 2 mL of culture through an airtight rubber seal. Aliquots were centrifuged, and the supernatant was stored at −80° and subsequently used for metabolite analysis. To select for live cells in the barcode microarray analysis of the homozygous and heterozygous deletion pools, pellets were resuspended in sterile water and grown on YPD + 200 µg/mL G418 agar (Difco, #DF0145170) medium at 25° for two days prior to harvesting and extraction of genomic DNA.

### Quantification of metabolites

Fermentation bottles were weighed every two days, and a sample of the supernatant was extracted for metabolite analysis. Ethanol, fructose, and glucose were detected using an Agilent 1100 Series HPLC system with an Agilent Refractive Index detector and autosampler. A 1 μL sample was injected onto a Supelcogel C610H cation exchange column and a Supelguard C610H guard column at 0.75 mL/min with a degassed 0.22 μm filtered 0.1% H_3_PO_4_ mobile phase using a column temperature of 50° and a refractive index temperature of 35°. Calibration and data analysis/quantitation were performed with LC/MSD Chemstation (Software Revision Rev. A.09.03). Yeast assimilable nitrogen (YAN; total alpha amino acids and ammonium) was measured with N-PANOPA and K-LARGE kits from Megazyme (Megazyme International Ireland) according to manufacturer’s instructions.

### Barcode amplification, hybridization, and image analysis

Barcode amplification and hybridization were performed as described (Cook *et al.* 2008) using amplification primers from Operon Biotechnologies. Arrays were scanned visually for anomalies, which were flagged and discarded as necessary. The median local background-subtracted intensities were converted to log2 ratios, and the data were Lowess normalized using the program Vector Xpression 3. Data with negative values in either channel were adjusted to a floor value of 1 for further calculations. Log2 intensity values [A = 0.5*log2(Cy5*Cy3)] were chosen individually for UP and DOWN (DN) barcode tags of each microarray to maximize recovery of true positives and minimize recovery of false positives as described (Cook *et al.* 2008) except using log2 intensity values instead of signal-to-noise ratios. In the case of the homozygous deletion pool experiments, intensity thresholds included less than 5% of false-positive signals. In the case of heterozygous deletion pool experiments, the majority of barcodes on the array were present within the pool. As such, an alternative method was required to set intensity thresholds; these were chosen qualitatively to exclude the low-intensity peak within the bimodal distribution of signals in a Log2 intensity (A) *vs.* frequency plot, representing presumptive nonfunctional barcodes. To further minimize false positives and maximize true positives, UP and DN barcodes with significant data for less than 40% of all replicate spots across all experiments were excluded from subsequent analysis. UP and DN tags for each array were independently converted to Z scores before being combined (centered to an average log2 ratio value of 0 and normalized to a standard deviation of 1). Replicate spots were averaged, and remaining false barcodes of strains not present in the homozygous or heterozygous deletion pool were removed. The data were clustered using Cluster 3.0 (De Hoon *et al.* 2004); prior to clustering, data were filtered to remove barcodes with no significant change across any time point (all log2 values < 1) or to include only those with significant change in 50% of experiments. Data were clustered hierarchically, with average linkage and an uncentered correlation similarity metric. Heat maps were generated using the program Java Treeview (Saldanha 2004). Microarray information can be accessed at ArrayExpress (E-MEXP-3332).

### Statistical calculations

To identify enriched functional categories, heterozygous and homozygous deletion mutants with a fitness disadvantage or advantage were queried using Funspec (Robinson *et al.* 2002), and categories with a *P* value of 0.01 or less were analyzed further. All genes listed under a given Munich Information Center for Protein Sequences (MIPS) category were compared with all genes from the homozygous deletion pool (3734) or the heterozygous deletion pool (5470) that had significant data on our array. The number of genes in each MIPS functional classification category (459 total categories were queried) that were present on the array are represented in Tables 2–5 in the “total genes in dataset” column. The number of genes with a 2-fold or greater decrease or increase in abundance compared with control in at least three of the fermentation time points are presented in the “total genes observed” column. *P* values were calculated using the hypergeometric cumulative distribution function and adjusted with a Bonferroni correction. Genes that were not annotated in the various MIPS categories, but nevertheless bear related functions, were manually added when necessary.

### Western blot analysis

The API Western blot was performed as follows. 1.5 × 10^7^ cells per sample were TCA precipitated, vortexed with 50 μL glass beads and 50 μL SDS gel loading buffer, boiled, run on an SDS-PAGE gel, and then transferred to nitrocellulose membrane. The membrane was probed with a 1:4000 dilution of anti-API antibody [kindly provided by Dr. Daniel Klionsky (Klionsky *et al.* 1992)] and a 1:5000 dilution of secondary antibody (goat-anti rabbit HRP conjugate, Bio-Rad).

### Electron microscopy

The homozygous *pep4Δ/pep4Δ* log phase and two-day fermented yeast were concentrated by centrifugation. The yeast were high-pressure frozen in Type A HPF specimen carriers (Technotrade International, Manchester, NH) with a Leica HPM 100 (Vienna, Austria). Frozen samples were then freeze-substituted in a Leica AFS (Vienna, Austria) as follows: two days at −85° in HPLC-grade acetone [containing 8% dimethoxypropane (DMP), 0.5% glutaraldehyde, 0.1% tannic acid], followed with several washes in clean acetone (−85°), transferred to HPLC-grade acetone (containing 1% osmium tetroxide and 0.1% uranyl acetate), held at −85° for two additional days, warmed to and held at −20° for 6 h, and finally warmed to room temperature. Substituted yeast were then washed in clean acetone and infiltrated with Spurr's resin over a graded series to 100% resin with rotation at room temperature. Ultrathin sections were cut using a Reichert Ultracut E ultramicrotome, picked up on formvar-coated 200-mesh copper grids, and stained with 2% uranyl acetate (aqueous) and Reynold’s lead citrate. Images were captured using an FEI Tecnai G2 (Hillsboro, OR) operated at 200 kV equipped with a 2K side-mounted Advanced Microscopy Techniques (AMT) digital camera (Danvers, MA).

## Results

### Identification of genes with a role in fermentation

To identify nonessential and essential *S. cerevisiae* genes required for optimal cellular fitness during fermentation, the S288C heterozygous and homozygous diploid yeast deletion mutant pools were individually grown in rich media to midlogarithmic phase, then diluted into synthetic grape juice (see *Materials and Methods*). The diploid yeast deletion mutant collections were chosen over the haploid collections because wine yeast are primarily diploid and because the diploid collections include both essential and nonessential genes. Anaerobic fermentations were carried out for 14 days at room temperature in 500-mL flasks equipped with a vapor lock. Fermentation profiles of the heterozygous and homozygous deletion mutant pools were plotted (supporting information, Figure S1). We carried out a fermentation of the industrial wine yeast strain EC1118 to demonstrate the fermentation profile of an industrial strain under the same conditions (Figure S1). Growth reached a maximal OD_600_ of ∼4.2 for both homozygous and heterozygous deletion pools, whereas the EC1118 wine yeast was able to grow to an OD_600_ of over 6.0 (Figure S1A). After 14 days, the concentration of ethanol in the homozygous and heterozygous diploid deletion pool fermentations was 12.4% and 12.0% (v/v), respectively, whereas the EC1118 fermentation attained 14.0% (v/v) ethanol (Figure S1B). As expected, the EC1118 fermentation was more robust than the S288C homozygous and heterozygous deletion mutant pools as ethanol production reached a plateau after 6 days of fermentation, whereas the S288C pools continued to produce ethanol over the entire 14-day period. There was no discernable difference between the fermentation profiles of the homozygous and heterozygous deletion set pools.

To avoid identification of yeast deletion strains that had fitness requirements due to the transfer of cells from rich media to synthetic grape juice, a control sample was taken after the homozygous and heterozygous deletion set pools had adapted for 24 h in synthetic grape juice, at which point the culture was still in logarithmic phase (Figure S1A). The day 1 time point was used as a hybridization control for all subsequent time points at days 2, 4, 6, 8, 10, and 14 of fermentation. Homozygous and heterozygous deletion strains that exhibited a 2-fold or greater alteration in barcode signal intensity in experimental over control sample in three or more time points were deemed to have either decreased or increased fitness during the fermentation. By this criterion, we identified 300 homozygous (Table S1) and 481 heterozygous (Table S4) deletion strains with reduced fitness during fermentation with an overlap of 38 genes (Table S7). Similarly, we identified 303 homozygous (Table S2) and 466 heterozygous (Table S5) deletion strains with increased fitness during fermentation with an overlap of 48 genes (Table S8). Of the heterozygous deletion mutants with reduced fitness, 116 are essential, and of the heterozygous deletion mutants with increased fitness, 111 are essential, which partially accounts for the low overlap between the datasets (Table 1). Approximately 20% of the deletion strains identified in each screen were deletions of uncharacterized genes and dubious ORFs (Table 1). Thus, nutrient and ethanol stress, as well as oxygen deprivation caused by fermentation, revealed potential roles for genes whose function has not been identified under conventional laboratory conditions.

**Table 1:** Summary of homozygous and heterozygous deletion set fermentation data

	Reduced Fitness		Increased Fitness	
	Heterozygous	Homozygous	Heterozygous	Homozygous
Total genes	481	300	466	303
Essential	116	0	111	0
Uncharacterized	52	24	38	32
Dubious	47	35	42	31

**Table 2:** Functional categories enriched in homozygous diploid mutants with a fitness disadvantage during fermentation

Functional Category*^a^*	Genes*^b^*	Total Genes Observed	Total Genes in Dataset	*P**^c^*
Autoproteolytic processing	ATG1, ATG2, ATG3, ATG5, ATG6/VPS30, ATG7, ATG8, ATG9, ATG10, ATG12, ATG13, ATG16, ATG18, ATG24/SNX4, ATG29*^d^*, ATG31/CIS1*^d^*, IRS4*^d^*, PEP4*^d^*, UTH1*^d^*	19	23	4.5 × 10^−17^
MIPS 14.07.11.01				
Modification by ubiquitination, deubiquitination	CUE1*^d^*, DOA1*^d^*, DOA10/SSM4, MMS2, RFU1*^d^*, RMD5*^d^*, SAN1, UBC5, UBC7, UBI4, UBP1, UBP13, UBP14, UBR2, UBX2*^d^*, UFD2, SKP2*^d^*	17	50	9.2 × 10^−6^
MIPS 14.07.05				

MIPS, Munich Information Center for Protein Sequences.

a Enrichment of functional categories was first defined according to Funspec (Robinson *et al.* 2002) with *P* ≤ 0.01. Additional genes were added manually.

b Genes listed are deletion mutants with a 2-fold or greater decrease in abundance compared with control in at least three of the fermentation time points.

c *P* value calculation in *Materials and Methods*.

d Manually annotated.

**Table 3:** Functional categories enriched in heterozygous diploid mutants with a fitness disadvantage during fermentation

Functional Category*^a^*	Genes*^b^*	Total Genes Observed	Total Genes in Dataset	*P**^c^*
Proteasome degradation (UPS)	APC1, CUE3*^d^*, ECM29*^d^*, HRD1, MDM30*^d^*, POC4*^d^*, PRE1, PRE2, PRE4, PRE7, PUP1, RPN5, RPT3, RPT5, RPT6, SLX8*^d^*, STS1, UBC5, UBP8*^d^*, UMP1, VMS1*^d^*, YDR306C, YLR224W	23	114	1.8 × 10^−2^
MIPS 14.13.01.01				

MIPS, Munich Information Center for Protein Sequences; UPS, ubiquitin-proteasome system.

a Enrichment of functional categories was first defined according to Funspec (Robinson *et al.* 2002) with *P* ≤ 0.01. Additional genes were added manually.

b Genes listed are deletion mutants with a 2-fold or greater decrease in abundance compared with control in at least three of the fermentation time points.

c *P* value calculation in *Materials and Methods*.

d Manually annotated.

**Table 4:** Functional categories enriched in homozygous diploid mutants with a fitness advantage during fermentation

Functional Category*^a^*	Genes*^b^*	Total Genes Observed	Total Genes in Dataset	*P**^c^*
Peroxisome	ANT1, PEX1, PEX4, PEX6, PEX8, PEX10, PEX13, PEX17, PEX22*^d^*, PIP2	10	31	7.5 × 10^−3^
MIPS 42.19				
Homeostasis of phosphate	MIR1, PHO84, PHO89, PHO91	4	7	2.9 × 10^−2^
MIPS 34.01.03.03				

MIPS, Munich Information Center for Protein Sequences.

a Enrichment of functional categories was first defined according to Funspec (Robinson *et al.* 2002) with *P* ≤ 0.01. Additional genes were added manually.

b Genes listed are deletion mutants with a 2-fold or greater increase in abundance compared with control in at least three of the fermentation time points.

c *P* value calculation in *Materials and Methods*.

d Manually annotated.

**Table 5:** Functional categories enriched in heterozygous diploid mutants with a fitness advantage during fermentation

Functional Category*^a^*	Genes*^b^*	Total Genes Observed	Total Genes in Dataset	*P**^c^*
Ribosomal proteins	DBP9, MRPL1, MRPL10, MRPL16, MRPS16, MRPS35, MRPS5, PET123, RPL10, RPL12A, RPL13B, RPL17B, RPL21A, RPL22A, RPL23B, RPL24A, RPL25, RPL26B, RPL2B, RPL34A, RPL36A, RPL40B, RPL41B, RPL6B, RPP1B, RPS0A, RPS12, RPS14B, RPS16A, RPS19A, RPS19B, RPS1B, RPS2, RPS22B, RPS23B, RPS24A, RPS24B, RPS26B, RPS29B, RPS31, RPS4A, RPS8A, RRP15, RSA3, RSM10, RSM23	46	198	1.5 × 10^−8^
MIPS 12.01.01				

MIPS, Munich Information Center for Protein Sequences.

a Enrichment of functional categories was first defined according to Funspec (Robinson *et al.* 2002) with *P* ≤ 0.01. Additional genes were added manually.

b Genes listed are deletion mutants with a 2-fold or greater increase in abundance compared with control in at least three of the fermentation time points.

c *P* value calculation in *Materials and Methods*.

d Manually annotated.

### Autophagy mutants have reduced fitness during fermentation

**Figure 1:**
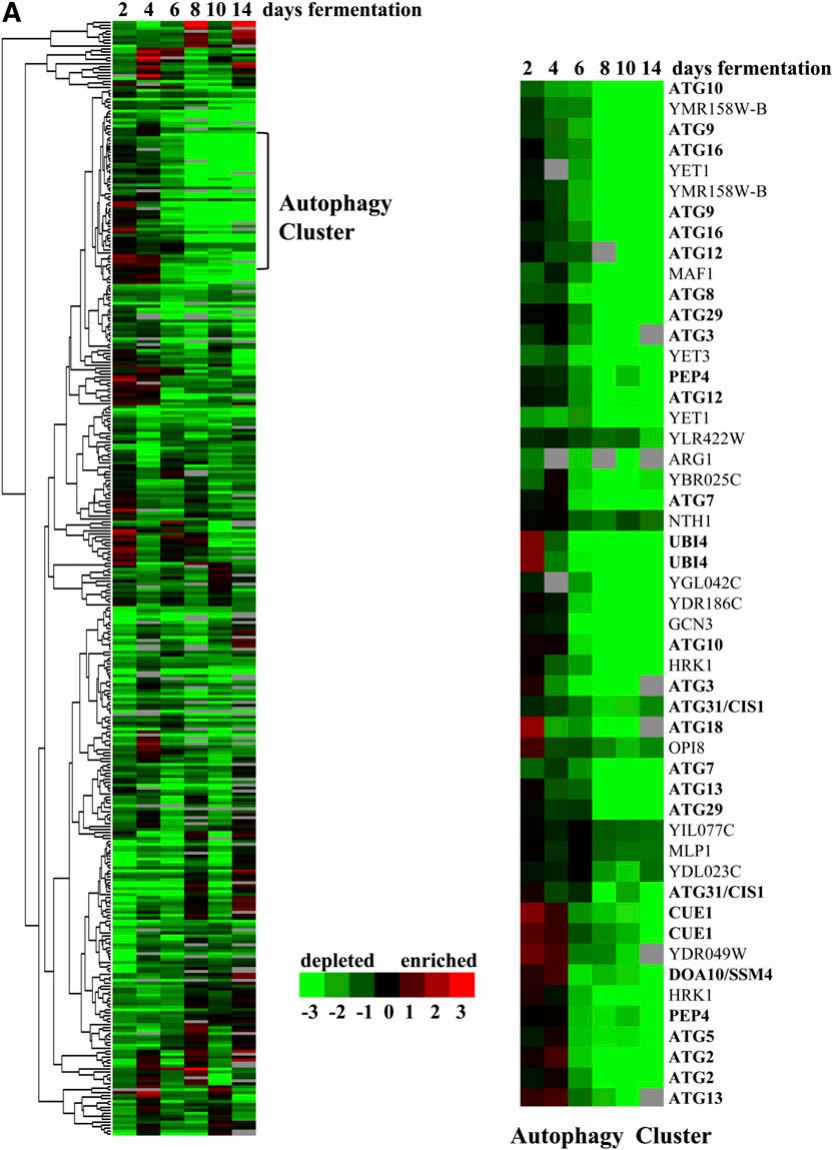

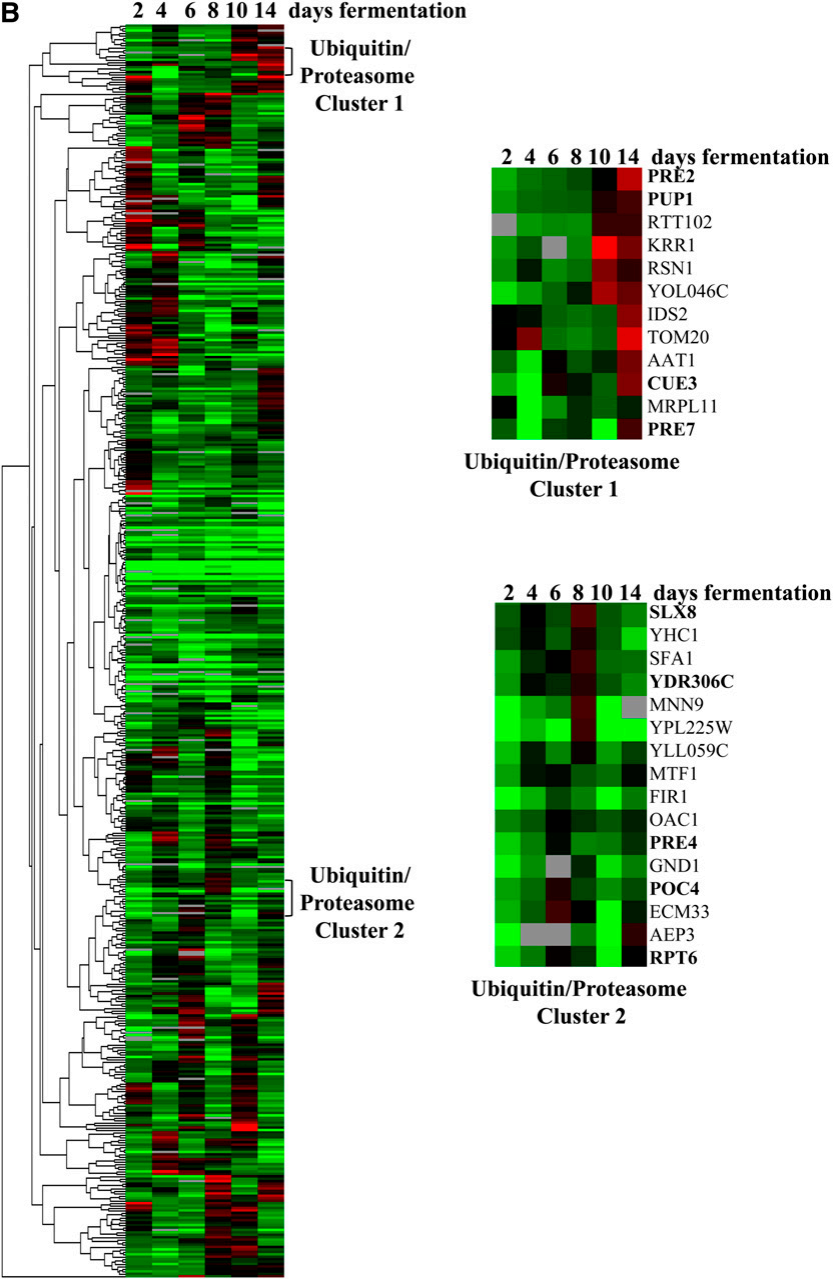
Hierarchical cluster analysis of (A) 300 homozygous and (B) 481 heterozygous diploid deletion strains with a fitness defect at 2, 4, 6, 8, 10, and 14 days of fermentation. Green, depletion; red, enrichment; black, no change; gray, no data. Numbers below the color bar represent the normalized log2 value of the microarray signal *vs.* day 1. Highlighted genes are in the functionally enriched categories of (A) autoproteolytic processing and modification by ubiquitination, deubiquitination, or (B) proteasome degradation. Genes that are listed twice on the clustergram have both UP and DOWN barcode tags that met the cutoff criteria.

**Figure 2:**
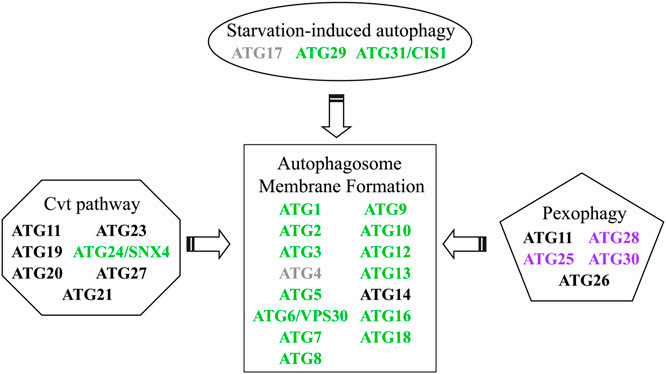
Core and starvation-induced autophagy genes contribute to cellular fitness during fermentation. The schematic is derived from Nakatogawa *et al.* (2009). Genes are color coded as follows: green, reduced fitness; black, no change in fitness; gray, no data obtained; purple, no homolog in *S. cerevisiae*.

We performed hierarchical cluster analysis of the 300 homozygous deletion strains (Figure 1A) and 481 heterozygous deletion strains (Figure 1B) with reduced fitness during fermentation. We found that some barcodes show inconsistencies across time points (*e.g.* depleted in day 2 yet enriched in day 4), likely due to noise in the data because each time point consisted of one biological replicate. Despite this caveat, clustering of the homozygous deletion strains with reduced fitness during fermentation revealed a strong cluster of autophagy genes, deletion of which caused progressive fitness defects over the fermentation period (Figure 1A). Consistently, autoproteolytic processing was the most highly enriched functional category in the entire dataset (*P* value of 4.5 × 10^−17^, Table 2). Autophagy is an evolutionarily conserved degradative process by which cytoplasmic constituents and organelles are sequestered into vesicles known as autophagosomes, which in turn fuse with the vacuole and thereby degrade their contents (Nakatogawa *et al.* 2009; Xie and Klionsky 2007). More selective forms of autophagy, in which specific organelles are engulfed and degraded, include the constitutively active cytoplasm-to-vacuole (Cvt) pathway, ribophagy, mitophagy, pexophagy, and reticulophagy (Kraft *et al.* 2009). A core set of 15 Atg proteins is required for autophagosome formation in all types of autophagy (Atg1-10, Atg12-14, Atg16,18) and is associated with the preautophagosomal structure (PAS), which is the site adjacent to the vacuole where autophagosomes are generated (Nakatogawa *et al.* 2009). Thirteen of 15 strains deleted for core *ATG* genes and 2 of the 3 strains deleted for starvation-induced autophagy genes were significantly depleted during fermentation (Figure 2, Table 2). In contrast, strains disrupted for 6 of the 7 *ATG* genes that have specific roles in the constitutively active Cvt pathway were not significantly selected for or against during fermentation (Figure 2, Table 2).

### Ubiquitin-proteasome pathway mutants have reduced fitness during fermentation

**Figure 3:**
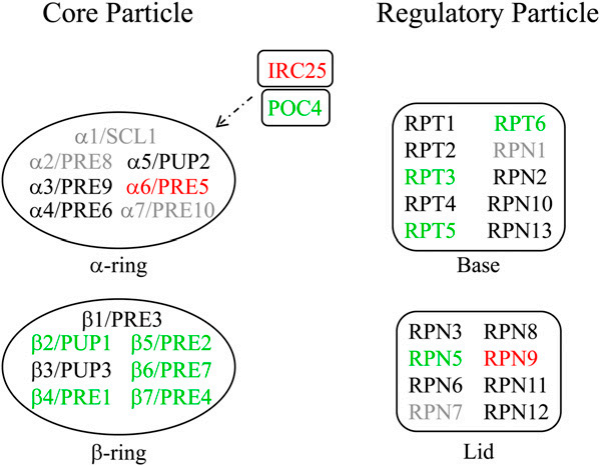
The β-ring of the proteasome core particle enhances cellular fitness during fermentation. The proteasome core particle α-ring (α1-7) and β-ring subunits (β1-7) are shown, along with the Irc25/Poc4 α-ring assembly chaperone. The proteasome regulatory particle (base and lid subunits) is also shown. Genes are color coded as follows: green, reduced fitness; red, increased fitness; black, no change in fitness; gray, no data obtained.

Cluster analysis of the heterozygous deletion strains that have decreased fitness during fermentation did not reveal a prominent cluster of genes as seen with the autophagy deletion strains; however, a functional enrichment query identified a significant enrichment of 23 genes with a role in the ubiquitin-proteasome system (UPS) (Figure 1B, Table 3). Indeed, we additionally observed that 17 homozygous strains deleted for ubiquitin-modification genes had overt fitness defects during fermentation, a significant enrichment of this functional class of genes (Table 2). The UPS is the major intracellular degradative pathway in eukaryotes whereby individual protein substrates are selectively conjugated to the small protein modifier ubiquitin by the action of a conserved cascade of E1 (ubiquitin-activating), E2 (ubiquitin-conjugating), and E3 (ubiquitin ligase) enzymes (Elsasser and Finley 2005; Hochstrasser 1996). Our analysis identified homozygous deletion strains disrupted in the stress-responsive ubiquitin gene (*UBI4*), ubiquitin-conjugating enzymes (*MMS2*, *UBC7*, *UBC5*), ubiquitin ligases (*DOA10/SSM4*, *RMD5*, *SAN1*, *SKP2*, *UBR2*), ubiquitin-specific proteases (*UBP1*, *UBP13*, *UBP14*), and other proteins that have various roles in the UPS (*CUE1*, *DOA1*, *RFU1*, *UBX2*, *UFD2*; see Table 2). Ubiquitylated proteins are targeted for degradation by the 26S proteasome, which is composed of a 20S core particle that contains the proteolytic active sites and a 19S regulatory particle that controls substrate entry (Finley 2009). We identified heterozygous deletion strains disrupted for components of the core particle (*PRE1*, *PRE2*, *PRE4*, *PRE7*, *PUP1*) and the regulatory particle (*RPN5*, *RPT3*, *RPT5*, *RPT6*), as well as proteins involved in proteasome assembly (*ECM29*, *POC4*, *UMP1*) and proteasome localization (*STS1*; Figure 1B, Figure 3, Table 3).

### Peroxisome biogenesis mutants have a fitness advantage during fermentation

**Figure 4:**
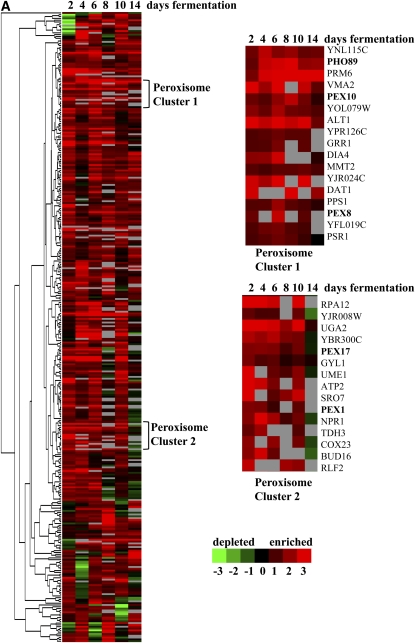

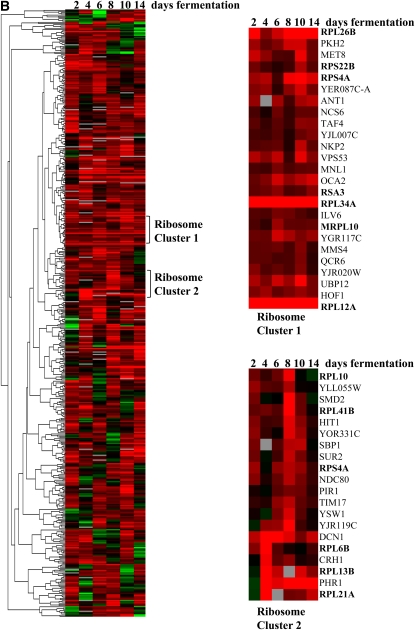
Hierarchical cluster analysis of (A) 303 homozygous and (B) 466 heterozygous diploid deletion strains with a fitness advantage at 2, 4, 6, 8, 10, and 14 days of fermentation. Green, depletion; red, enrichment; black, no change; gray, no data. Numbers below the color bar represent the normalized log2 value of the microarray signal *vs.* day 1. Highlighted genes are in the functionally enriched categories of (A) peroxisome or (B) ribosomal proteins.

Hierarchical cluster analysis of the 303 homozygous deletion strains that have increased fitness during fermentation did not reveal a prominent cluster of genes; however, a functional enrichment query identified a significant enrichment for peroxisome biogenesis genes (Figure 4A, Table 4). The biogenesis of peroxisomes requires the posttranslational import of peroxisomal matrix proteins; this process requires recognition by a receptor/docking complex at the surface of the peroxisome, translocation across the peroxisomal membrane, release into the peroxisome matrix, and recycling of the receptor (Smith and Aitchison 2009). Deletion strains defective in the docking complex (*PEX13*, *PEX17*), translocation process (*PEX8*), and receptor recycling (*PEX1*, *PEX4*, *PEX6*, *PEX10* and *PEX22*) all had increased fitness during fermentation (Table 4). A strain deleted for a dubious ORF (*YJL211C*) that overlaps with the *PEX2* receptor recycling gene was also enriched during fermentation (Table S2).

### Phosphate transport mutants are beneficial for cellular fitness during fermentation

A second functional category of homozygous deletion strains that was enriched during fermentation included disruptions of genes with roles in phosphate homeostasis. Strains lacking high-affinity phosphate transporters (*PHO84*, *PHO89*), a low-affinity phosphate transporter (*PHO91*), and the *MIR1* mitochondrial phosphate carrier were all enriched as fermentation proceeded (Table 4). When cells are starved of phosphate, the expression of *PHO84*, *PHO89*, and the secreted acid phosphatases (*PHO5*, *PHO11*, *PHO12*) is induced to facilitate the scavenging of phosphate (Mouillon and Persson 2006). When phosphate is plentiful, Pho84 is removed from the plasma membrane and targeted to the vacuole for degradation (Lagerstedt *et al.* 2002). Similarly, Pho91 and another low-affinity phosphate transporter, Pho87, are targeted for endocytosis and vacuolar degradation by ubiquitination (Estrella *et al.* 2008). As our synthetic grape juice medium was not limiting for phosphate, we speculate that unnecessary phosphate transport expression and recycling may actually confer a fitness disadvantage under these conditions.

### Ribosomal mutants have a fitness advantage during fermentation

Functional enrichment analysis of heterozygous deletion mutants with a fitness advantage during fermentation revealed a significant enrichment for the functional category of ribosomal proteins (*P* value of 1.5 × 10^−8^, Table 5). Hierarchical cluster analysis was performed and two clusters of ribosomal genes were identified, although not all 46 ribosomal genes mapped to these clusters (Figure 4B). We identified heterozygous deletion mutants in components of the small (*RPS*) and large (*RPL*) ribosomal subunits; the ribosomal stalk (*RPP1B*); and components of the mitochondrial small (*MRPS*, *PET123*, *RSM*) and large (*MRPL*) ribosomal subunits, as well as proteins involved in ribosome biogenesis (*DBP9*, *RRP15*, *RSA3*). As deletion of some ribosomal proteins and known ribosome biogenesis factors did not appear to alter fitness during fermentation, it may be that the deletion strains that conferred resistance correspond to ribosome synthesis pathways that integrate specific aspects of nutrient responsiveness. Differential effects of ribosome proteins and ribosome biogenesis gene deletions have been observed previously in cell-size screens (Jorgensen *et al.* 2002).

### Deletion mutants with altered fitness in three sequential fermentation time points are still enriched for the same functional categories

The cellular environment changes rapidly during fermentation due to the consumption of nutrients and production of ethanol. Thus, genes may be required at a specific stage of fermentation but not throughout the entire fermentation. Therefore, we reanalyzed our data in a highly stringent manner by requiring that deletion mutants display a 2-fold or greater alteration in barcode signal intensity in experimental over control sample in three or more sequential time points (Table S9, Table S10, Table S11, Table S12). By these criteria, we identified 180 homozygous (Table S9) and 210 heterozygous (Table S11) deletion mutants with reduced fitness during fermentation with an overlap of 20 genes (Table S13). One hundred sixty-two homozygous (Table S10) and 214 heterozygous (Table S12) deletion mutants have increased fitness during fermentation in three consecutive time points with an overlap of 18 genes (Table S14). We performed analysis of functional enrichment within each new dataset and found that all of the categories that were enriched previously (Tables 2–5) were still enriched with the sequential datasets, with the exception of the peroxisome genes (Table 4). This finding is likely due to data points that were missing from a few of the peroxisome genes (*e.g.*, *PEX1* and *PEX8* in Figure 4A). However, the overlap between the homozygous and heterozygous mutants with a fitness advantage during fermentation (Table S8) is enriched for genes with a role in fatty acid oxidation, which occurs in the peroxisome (see *Discussion*).

### Autophagy mutants have reduced CO_2_ and ethanol production during fermentation

**Figure 5:**
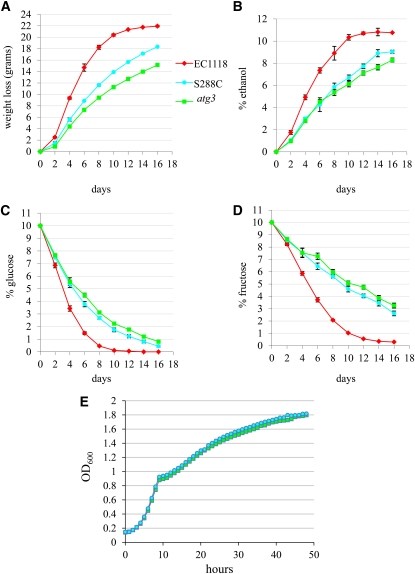
Disruption of *atg3* causes reduced CO_2_ and ethanol production during fermentation. EC1118 (red diamonds), wild-type (S288C, light blue circles), and *atg3Δ* (green squares) homozygous diploid strains were inoculated into synthetic grape juice supplemented with amino acids, and anaerobic fermentation was carried out for 16 days. Weight loss (A), ethanol (B), glucose (C), and fructose (D) measurements were taken every 2 days. Each data point on the graph represents the average of two EC1118, four wild-type, and four *atg3Δ* fermentations. For all graphs, error bars represent the standard deviation for each data point. (E) Growth chamber analysis of wild-type (S288C) *vs.* *atg3Δ* strain growth in YPD at 25°. Cells were inoculated into a multiwell plate at an OD_600_ of 0.1 and grown for 50 h. Each data point is an average of three technical replicates. For each data point, SD < ±0.04.

Of the genes required for cellular fitness during fermentation, we chose to focus our biological studies on the autophagy pathway because it was the most enriched functional category (Table 2) and revealed a strong cluster of genes based on hierarchical cluster analysis (Figure 1A). We directly measured the effects of *ATG* gene disruptions on fermentation parameters by performing 16-day fermentations with seven different diploid *atg* homozygous deletion strains compared with the S288C wild-type diploid parental strain and the EC1118 industrial wine yeast strain. The *atg* deletion strains exhibited highly similar fermentation profiles; therefore, a representative example (*atg3Δ*) is shown (Figure 5). As expected, EC1118 had a higher rate of fermentation than S288C with 22.0 g final weight loss and 10.8% (v/v) ethanol produced (Figure 5A, B). Compared with the S288C parental strain, *atg3Δ* strains had a slower rate of weight loss, indicating lower CO_2_ production and a reduced fermentation rate (Figure 5A). The final weight loss for the S288C parental strain was 18.4 g compared with 15.2 g for *atg3Δ* strains. Similarly, the final level of ethanol produced after the 16-day fermentation by the S288C parental strain was 9.0% (v/v) compared with 8.3% (v/v) for *atg3Δ* (Figure 5B). All of the glucose and most of the fructose (99.7%) were depleted in the EC1118 fermentation. However, the S288C fermentation contained 0.4% glucose and 2.6% fructose, and the *atg3Δ* fermentation contained 0.8% glucose and 3.3% fructose after 16 days (Figure 5C, D). In all cases, the differences between wild-type and *atg3Δ* strains are statistically significant (*P* < 0.02, unpaired Student’s *t*-test). The *atg3Δ* strains do not have a slow growth phenotype compared with wild-type cells grown in rich media; therefore, lower production of ethanol by *atg3Δ* cells during fermentation cannot be attributed simply to attenuated growth (Figure 5E). These results demonstrate that in addition to competitive fitness defects at the population level under fermentation conditions, disruption of autophagy genes causes cells to ferment at a slower rate, resulting in reduced ethanol output and residual sugars.

### The autophagy pathway is functional during fermentation

**Figure 6:**
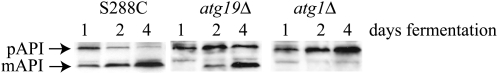
API processing occurs via autophagy during fermentation. Western blot analysis of cell lysates from wild-type diploid (S288C) and *atg19Δ* and *atg1Δ* homozygous diploids at days 1, 2, and 4 of fermentation. Blots were probed with anti-API antibody. Arrows point to the precursor form (pAPI) and the mature form (mAPI) of API. API, aminopeptidase I.

Our fitness profiling data suggested that autophagy, but not the constitutively active Cvt pathway, is required for optimal fitness during fermentation (Figure 2). The yeast vacuolar hydrolase aminopeptidase I (API) is synthesized in the cytoplasm in a precursor form (pAPI) that is targeted to the vacuole by either the Cvt pathway (logarithmic growth) or the autophagy pathway (starvation) (Baba *et al.* 1997; Klionsky *et al.* 1992). The conversion of pAPI to its mature form (mAPI) is associated with transport of pAPI to the vacuole and requires the Atg19 receptor in the Cvt pathway (Leber *et al.* 2001; Scott *et al.* 2001). However, upon prolonged nitrogen starvation, pAPI is processed to mAPI in the absence of Atg19 by the autophagy pathway (Leber *et al.* 2001; Scott *et al.* 2001). We performed a fermentation with wild-type (S288C) and *atg19* and *atg1* deletion mutants and monitored conversion of pAPI into mAPI. As expected, we detected mAPI in wild-type cells at the start of the fermentation (day 1), and the majority of pAPI was processed by day 4 of fermentation (Figure 6). pAPI was also processed to mAPI in *atg19* mutants, albeit at a slower rate, which was previously shown under conditions of prolonged starvation (Leber *et al.* 2001) (Figure 6). However, no processing of pAPI was observed in *atg1* mutants because Atg1 is essential for pAPI processing under all conditions (Figure 6). As pAPI is converted to mAPI in an Atg19-independent, but Atg1-dependent, manner, the autophagy pathway is responsible for the transport of Atg19 to the vacuole during fermentation, suggesting that autophagy is occurring during fermentation.

### Autophagy is induced during fermentation

**Figure 7:**
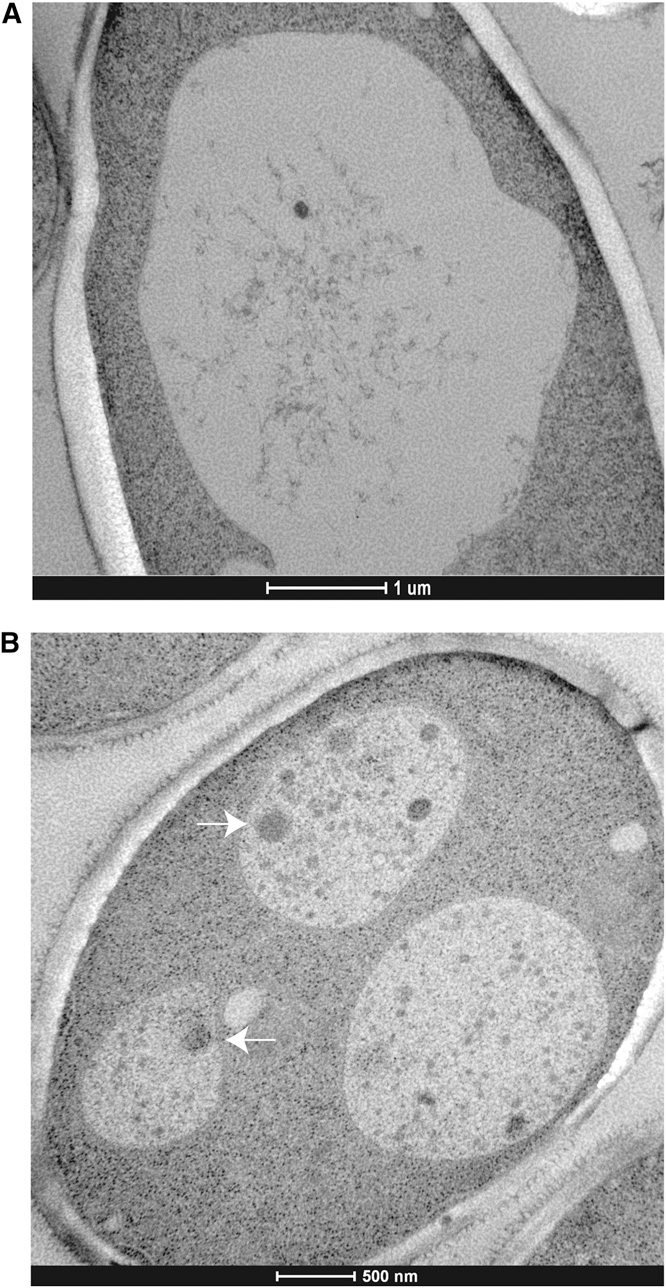
Autophagic bodies accumulate in the vacuole during fermentation. Electron microscopy of *pep4Δ/pep4Δ* homozygous diploid log phase cells (A) and after two days of fermentation (B). Arrows point to autophagic bodies in the vacuole.

To demonstrate that autophagy is induced during fermentation, we performed electron microscopy (EM) analysis with *pep4* homozygous mutant cells after two days of fermentation compared with log phase *pep4* homozygous mutant cells. Autophagic bodies are rapidly degraded in wild-type cells upon entry into the vacuole, thus necessitating a vacuolar proteinase-deficient strain (such as a *pep4* mutant) to visualize them (Takeshige *et al.* 1992). Of the vacuoles of *pep4* log phase cells (N = 26), 70% were devoid of large membrane-bound vesicles (Figure 7A), whereas after two days of fermentation, autophagic bodies were clearly detected in 75% of the vacuoles imaged (N = 20, Figure 7B, arrows). Autophagic bodies are typically 300–900 nm in diameter compared with Cvt vesicles, which are much smaller (∼150 nm) (Baba *et al.* 1997; Baba *et al.* 1994; Takeshige *et al.* 1992). In addition to the autophagic bodies, we detected smaller vesicles in *pep4* mutants after two days of fermentation, suggesting that the Cvt pathway may also be active (Figure 7B). Alternatively, the vacuolar sap could contain remnants of broken down autophagic bodies (Takeshige *et al.* 1992). Vacuoles are known to fragment when exposed to osmotic stress, and our EM images suggested that fermentation conditions may also induce vacuole fragmentation [(Bonangelino *et al.* 2002), Figure 7B]. Using a vacuolar-specific stain, we confirmed that vacuoles are indeed fragmented during fermentation (Figure S2). These data suggest that both autophagy and rearrangement of the vacuole are induced during wine fermentation.

### Autophagy is induced in the presence of nitrogen

**Figure 8:**
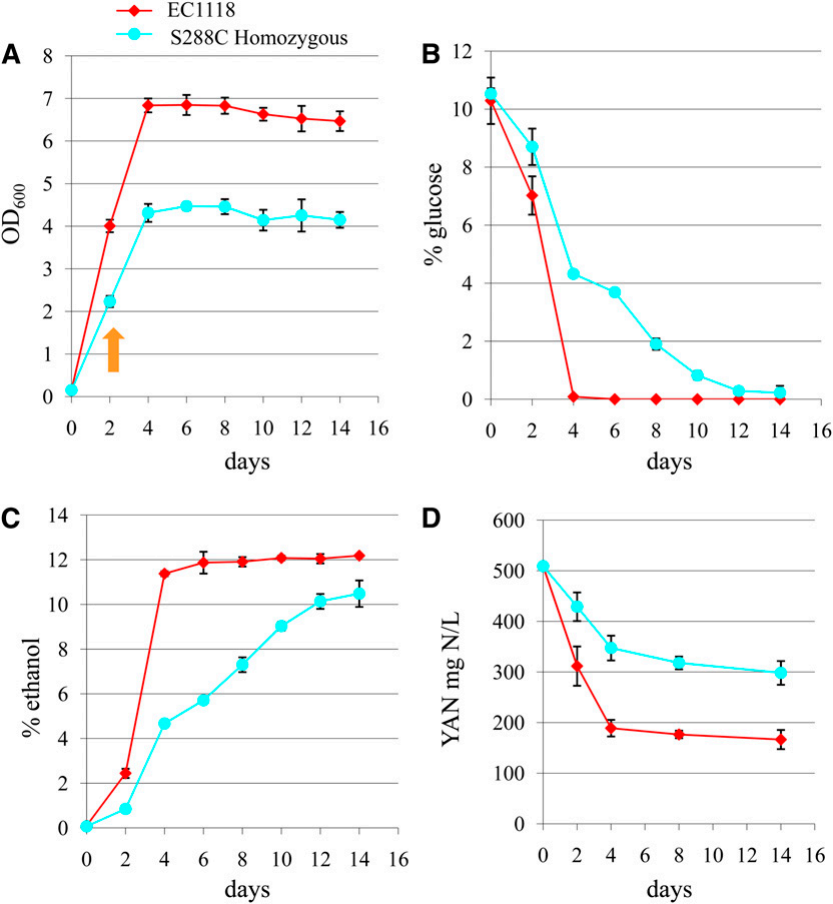
Fermentation kinetics of the homozygous yeast deletion pool (S288C) compared with the EC1118 wine yeast strain in synthetic grape juice. Both strains [EC1118 (red diamonds) and diploid homozygous deletion set (S288C, light blue circles)] were fermented in triplicate. The average values are presented with error bars representing SD. (A) Cell growth curve measured by OD_600_, (B) glucose depletion, (C) ethanol production, and (D) yeast assimilable nitrogen [YAN, milligrams of nitrogen (N) per liter] measured from total ammonium sulfate and amino acids. Orange arrow in (A) marks when autophagic bodies are detected by electron microscopy. YAN, yeast assimilable nitrogen.

The target of rapamycin (Tor) and protein kinase A-Sch9 signaling pathways negatively regulate autophagy; induction of autophagy requires release from this inhibition via activation of the Atg1-Atg13 kinase complex (Kamada *et al.* 2010; Noda and Ohsumi 1998; Stephan *et al.* 2009; Yorimitsu *et al.* 2007). Indeed, we find that a homozygous *tor1Δ* strain is enriched during fermentation suggesting that release from Tor1-mediated autophagy inhibition is beneficial for fermentation (Table S2). Induction of autophagy in yeast has been well studied under conditions of nitrogen starvation and inhibition of Tor under starvation conditions induces autophagy (Nakatogawa *et al.* 2009). To determine if nitrogen pools are depleted during our fermentation study, we did a careful analysis of the homozygous deletion set fermentation compared with the EC1118 wine yeast strain by monitoring total YAN along with metabolites, growth, and weight loss measurements. After 14 days of fermentation, the concentration of ethanol in the homozygous diploid deletion pool fermentation was 10.5% (v/v) compared with 12.2% (v/v) for EC1118 (Figure 8C). Glucose was depleted by 4 days in the EC1118 strain and by 12 days in the S288C pool (Figure 8B). Notably, total YAN, which is a measurement of total ammonium sulfate and amino acids, was retained in all fermentations, suggesting that global nitrogen starvation did not occur (Figure 8D).

Although our YAN measurements suggest that autophagy was not induced by global nitrogen starvation, we cannot assess whether single amino acids have been depleted from the media using this method. Depletion of single amino acids has been demonstrated to induce autophagy, and although we added amino acids to our fermentation media to address auxotrophies in the deletion set, single amino acid depletion may have occurred (Takeshige *et al.* 1992). However, autophagy is induced by two days of fermentation when cells are still in logarithmic growth (Figure 8A, orange arrow). To verify the presence of all auxotrophically required amino acids, we performed a fermentation with the homozygous deletion set parental strain, and on day two, we filtered the yeast from the fermentation and reinoculated the juice with log phase cells at an OD_600_ of 0.2. The media isolated from day two of fermentation, when autophagic bodies can be detected, enabled the newly inoculated cells to grow to an OD_600_ of over 2.0, suggesting that none of the auxotrophic amino acids were depleted (data not shown). On the basis of these results, we propose that autophagy during fermentation is not induced by amino acid depletion.

## Discussion

Our genome-wide survey of the genetic requirements in *S. cerevisiae* for wine fermentation reveals a primary function for autophagy in this process (Figure 1A, Table 2). The recycling of cellular components by autophagy enables yeast to survive the stressful conditions of fermentation and maximize fermentative output. Consistent with our functional profiles, gene expression studies of *S. cerevisiae* wine strains reveal that the autophagy genes *ATG1*, *ATG8*, and *PEP4* are induced during fermentation (Marks *et al.* 2008; Rossignol *et al.* 2003). In proteomic studies, protein fragments of glycolytic enzymes that are proteolysed in the vacuole have been detected during fermentation (Trabalzini *et al.* 2003). Although autophagy has not previously been documented during a primary yeast fermentation, it has been shown that autophagy is induced during secondary fermentation, possibly as a prelude to yeast cell autolysis (Cebollero and Gonzalez 2006; Cebollero *et al.* 2008). Together with these previous studies, our data suggests that autophagy enables yeast to survive the harsh nutrient and stress conditions that accompany fermentation. We find that autophagy is induced in the presence of nitrogen and sufficient amino acids to support doubling of cells, suggesting that an alternative signal may trigger autophagy induction during fermentation (Figures 6–8).

Our analysis revealed that defects in peroxisome biogenesis and ribosome assembly confer a significant fitness advantage during fermentation (Figure 4, Tables 4, 5). Peroxisomes are degraded by a specialized form of autophagy, termed pexophagy, which is induced upon shift from oxidative growth on poor carbon sources to fermentative growth on glucose (Sakai *et al.* 2006). The enrichment of strains defective in peroxisome biogenesis raises the possibility that pexophagy is a critical autophagic pathway that is triggered under fermentative conditions. Interestingly, phosphofructokinase protein, but not its enzymatic activity, is required for glucose-induced pexophagy in the methylotrophic yeast *P. pastoris* (Yuan *et al.* 1997). One intriguing possibility is that increased glycolytic protein abundance under fermentation conditions is a trigger for pexophagy. We identified 48 genes that overlap between the homozygous and heterozygous deletion mutants with increased fitness during fermentation that are enriched for the MIPS functional classification of oxidation of fatty acids (Table S8, Robinson *et al.* 2002). One of these genes, *PIP2*, which encodes the Pip2-Oaf1 transcription factor complex that induces peroxisomal gene expression in response to fatty acids, is repressed by glucose (Gurvitz and Rottensteiner 2006). Two deletion strains with defects in fatty acid β-oxidation (*ANT1* and *TES1*), which takes place in the peroxisome in yeast, also had a fitness advantage during fermentation in both the homozygous and heterozygous datasets (Table S8). In addition, a homozygous deletion of the dubious ORF *YNL203C*, which removes most of the overlapping *SPS19* fatty acid β-oxidation gene, was enriched during fermentation (Table S2). These observations suggest that peroxisome-mediated catabolism may adversely affect strain viability during fermentation.

Ribosomes are degraded by a specialized form of autophagy termed ribophagy (Kraft and Peter 2008; Kraft *et al.* 2010). Ribosomes constitute about half of the total cellular protein, therefore their degradation may be a major amino acid source under nutrient-limiting conditions such as fermentation. Both ribosome assembly and protein translation consume energy, so downregulation of these processes might enable cell survival under nutrient-limited conditions. Indeed, we identified a number of heterozygous mutants in genes involved in tRNA synthesis that were enriched during fermentation, supporting this hypothesis (Table S5). Recent studies have implicated the Ubp3 ubiquitin protease, its cofactor Bre5, and the Rsp5 ubiquitin ligase in ribophagy (Kraft *et al.* 2008; Kraft and Peter 2008; Kraft *et al.* 2009). An intriguing possibility is that some of the ubiquitin-modifying proteins identified in our study may have direct roles in selective autophagy during fermentation.

We found that the 26S proteasome is required for fermentation fitness, suggesting that the UPS is important in the adaptive response to fermentation. In agreement with our data, the single ubiquitin encoding gene in yeast, *UBI4*, is induced during starvation, and overexpression of *UBI4* confers ethanol tolerance (Chen and Piper 1995; Finley *et al.* 1987; Fraser *et al.* 1991). In addition, *UBI4* was identified from a previous small-scale competitive growth screen as being required for stress tolerance during ethanol production (Sharma *et al.* 2001). This result and the fitness defect of strains disrupted for ubiquitin-specific proteases suggest that ubiquitin itself may be limiting during the extensive proteome and membrane remodeling that occurs during fermentation.

The 20S core particle of the 26S proteasome forms a barrel with a stack of four 7-membered rings (2 inner rings formed by β-type subunits and 2 outer rings formed by α-type subunits) (Finley 2009). We identified heterozygous mutants of five β-type subunits (*PRE1*, *PRE2*, *PRE4*, *PRE7*, *PUP1*) that had a fitness disadvantage during fermentation (Table 3, Figure 3). In contrast, a heterozygous deletion strain of *PRE5*, the α6 subunit of the proteasome, had a fitness advantage during fermentation (Figure 3, Table S5). The β-type subunits contain the proteolytic-active sites of the proteasome, which could explain why loss of these subunits is more detrimental to cellular fitness during fermentation (Finley 2009). Assembly of a specific isoform of the 20S core particle is regulated by the Pba3/Irc25–Pba4/Poc4 assembly chaperone (Kusmierczyk *et al.* 2008). Unexpectedly, we detected a fitness defect for the *pba4/poc4* heterozygous deletion mutant but a fitness advantage for the *pba3/irc25* heterozygous deletion mutant (Figure 3, Table 3, Table S4, Table S5). One intriguing possibility is that alternative forms of the 20S core particle enhance cell fitness during fermentation.

Our fitness profiles also revealed a number of other genes with discrete functions that are necessary for optimal growth and survival during fermentation (Table S1, Table S2, Table S3, Table S4, Table S5). In particular, our profiles assigned fermentative phenotypes to 139 uncharacterized genes and 143 dubious ORFs. Most of the dubious ORFs overlap genes with known function, some of which are implicated in autophagy, proteasomal degradation, and ribosome and peroxisome biogenesis. For example, the dubious ORF *YMR158W-B*, the deletion of which was selected against during fermentation (Figure 1A, Table S1), overlaps with the autophagy gene *ATG16*. Similarly, the dubious ORF *YJL211C*, the deletion of which was enriched during fermentation (Table S2), overlaps with the peroxisomal gene *PEX2*, and the dubious ORF *YLR339C*, which was enriched in the heterozygous deletion set data, overlaps the essential ribosomal protein *RPPO* (Table S5). The roles of other uncharacterized genes in fermentation remain to be determined.

A heterozygous deletion mutant fitness profiling study was previously reported in nutrient-limiting conditions, including grape juice media (Delneri *et al.* 2008). We compared our fermentation fitness profiling data to the deletion mutants identified as haploinsufficient (decreased growth rate) and haploproficient (increased growth rate) from the Delneri *et al.* (2008) dataset. Heterozygous deletion mutants with a fitness disadvantage during fermentation displayed a statistically significant, albeit modest, 2-fold greater overlap than expected by chance with haploinsufficient mutants grown in carbon-limiting, nitrogen-limiting, and grape juice media (Figure S3). With the exception of a minor enrichment in carbon-limiting conditions, no significant overlap was detected between mutants with a fitness advantage during fermentation and haploproficient mutants grown in nutrient-limiting media (Figure S3). One possibility for this lack of overlap is that we performed a closed fermentation under anaerobic conditions, whereas Delneri *et al.* (2008) grew their mutant pool in continuous cultures in aerobic conditions. The difference in environmental conditions could be why UPS mutants were haploproficient in nitrogen-limiting conditions, whereas we found that UPS mutants had a fitness disadvantage during fermentation. Delneri *et al.* (2008) postulated that “protein conservation may be beneficial” under nitrogen-limiting conditions, whereas we postulate that protein recycling is beneficial to fermenting cells. We performed an analysis of functional enrichment within the Delneri *et al.* (2008) datasets and found that, similar to our heterozygous fermentation data, ribosomal proteins were haploproficient in carbon-source–limiting media (Table 5). Therefore, both studies suggest that a decrease in protein production is beneficial to cells under nutrient stress. Indeed, slowing the growth rate of ribosomal mutants in minimal media rescues their growth defect in rich media (Deutschbauer *et al.* 2005).

The comprehensive identification of *S. cerevisiae* gene function has been hampered to some extent by the use of standard laboratory growth conditions. By subjecting the laboratory yeast deletion strain collection to harsh fermentation conditions, we have uncovered the cellular processes and gene function processes necessary for fitness in this natural environment. The identification of genes required for cellular fitness during fermentation should facilitate the genetic engineering of wine yeast strains that are hypertolerant to the harsh conditions of industrial wine fermentation. Recent global transcription machinery engineering (gTME) demonstrates the feasibility of constructing strains with superior fermentative capacity (Alper *et al.* 2006). Notably, we identified 33 transcription factor genes that modulate fitness during fermentation (Table S1, Table S2, Table S3, Table S4, Table S5); these factors are candidates for future gTME efforts to create specialized fermentation strains. Similarly, the manipulation of autophagy, peroxisome biogenesis, ubiquitin-dependent protein modification, metabolism, and other processes may enhance the utility of yeast in food, enzyme, and biofuel production.

## Supplementary Material

Supporting Information
