# Supplementary material for: Genome-wide Fitness Profiles Reveal a Requirement for Autophagy During Yeast Fermentation
Source: G3 (Bethesda). 2011 Oct 1;1(5):353–67. doi: 10.1534/g3.111.000836 (PMC3276155; doi:10.1534/g3.111.000836)
Supplement: Supporting Information [file supp_1_5_353__index.html]

Supporting Information 

# Genome-wide Fitness Profiles Reveal a Requirement for Autophagy During Yeast Fermentation

## Supporting Information for Piggot *et al.*, 2011

**Files in this Data Supplement:**

- Supporting Information - Figures S1-S3 and Tables S1-S14 (PDF, 528 KB)
- Figure1 - Fermentation of the homozygous and heterozygous yeast deletion pools (S288C) and the EC1118 wine yeast strain in synthetic grape juice (PDF, 60 KB)
- Figure S2 - Vacuoles fragment during fermentation (PDF, 60 KB)
- Figure S3 - Heterozygous deletion mutants with decreased fitness during fermentation are enriched for mutants with reduced growth rates in nutrient limiting conditions (PDF, 340 KB)
- Table S1 - Homozygous deletion mutants with reduced fitness during fermentation (.xlsx, 20 KB)
- Table S2 - Homozygous deletion mutants with increased fitness during fermentation (.xlsx, 20 KB)
- Table S3 - Homozygous deletion mutant pool fermentation fitness profiles (.xlsx, 1.6 MB)
- Table S4 - Heterozygous deletion mutants with reduced fitness during fermentation (.xlsx, 24 KB)
- Table S5 - Heterozygous deletion mutants with increased fitness during fermentation (.xlsx, 24 KB)
- Table S6 - Heterozygous deletion mutant pool fermentation fitness profiles (.xlsx, 1.1 MB)
- Table S7 - Overlap between homozygous and heterozygous deletion mutants with reduced fitness during fermentation (.xlsx, 12 KB)
- Table S8 - Overlap between homozygous and heterozygous deletion mutants with increased fitness during fermentation (.xlsx, 12 KB)
- Table S9 - Homozygous deletion mutants with reduced fitness during fermentation in three or more sequential time points (.xlsx, 16 KB)
- Table S10 - Homozygous deletion mutants with increased fitness during fermentation in three or more sequential time points (.xlsx, 16 KB)
- Table S11 - Heterozygous deletion mutants with reduced fitness during fermentation in three or more sequential time (.xlsx, 16 KB)
- Table S12 - Heterozygous deletion mutants with increased fitness during fermentation in three or more sequential time points (.xlsx, 16 KB)
- Table S13 - Overlap between homozygous and heterozygous deletion mutants with reduced fitness during fermentation in three or more sequential time points (.xlsx, 12 KB)
- Table S14 - Overlap between homozygous and heterozygous deletion mutants with increased fitness during fermentation in three or more sequential time points (.xlsx, 12 KB)
