# Supplementary material for: Genome-wide Fitness Profiles Reveal a Requirement for Autophagy During Yeast Fermentation
Source: G3 (Bethesda). 2011 Oct 1;1(5):353–67. doi: 10.1534/g3.111.000836 (PMC3276155; doi:10.1534/g3.111.000836)
Supplement: Supporting Information [file supp_1.5.353_000836.pdf]

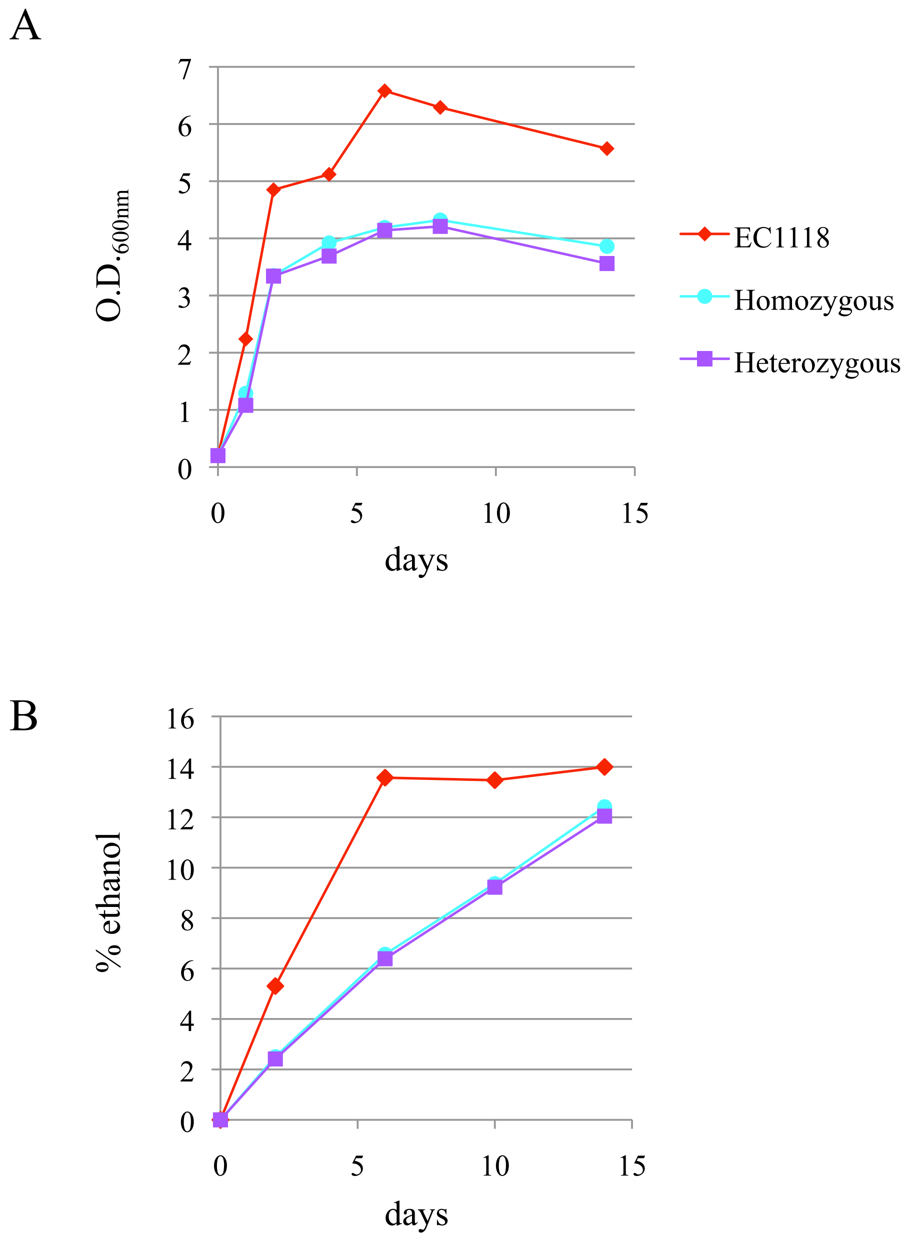

Figure S1 Fermentation of the homozygous and heterozygous yeast deletion pools (S288C) and the EC1118 wine yeast strain in synthetic grape juice. Samples extracted from these fermentations were used for genome-wide fitness profiling. (A) Cell growth curve measured by $O.D._{600nm}$, (B) ethanol production.

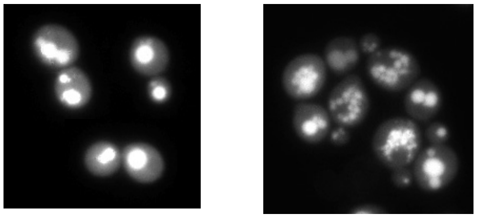

Figure S2 Vacuoles fragment during fermentation. Diploid wild type yeast cells were imaged during log phase growth and after one day of fermentation in synthetic grape juice. Vacuoles were stained with the vacuole-specific dye CMAC (7-amino-4-chloromethylcoumarin) and imaged using a fluorescence microscope.

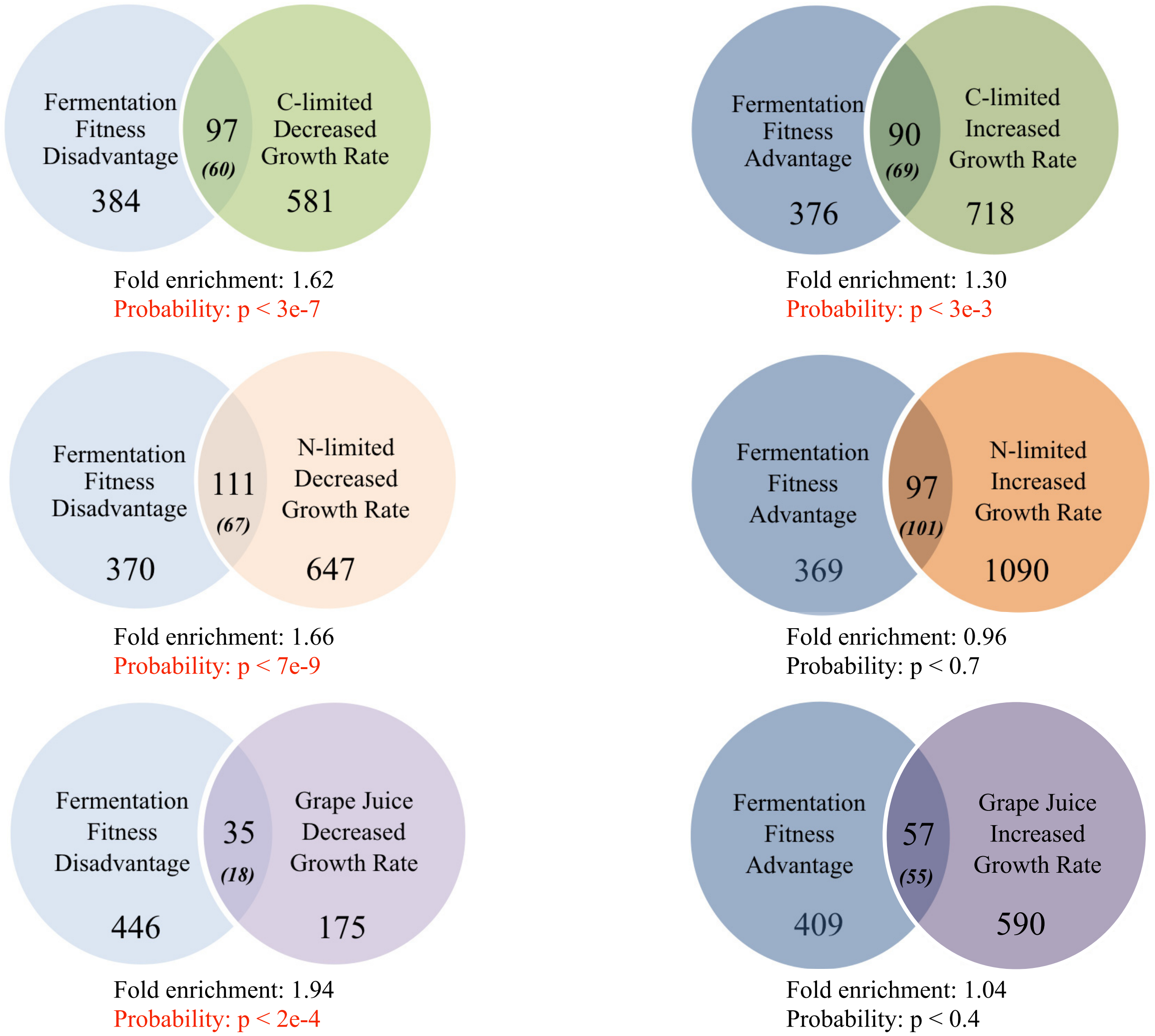

Figure S3 Heterozygous deletion mutants with decreased fitness during fermentation are enriched for mutants with reduced growth rates in nutrient limiting conditions. The heterozygous deletion mutants with reduced or increased fitness during fermentation were compared to mutants with decreased or increased growth rate in nitrogen-limiting (N-limited), carbon-limiting (C-limited) and grape juice media (Delneri *et al.* 2008). The unique genes in each dataset as well as the overlap are displayed using a Venn diagram. The amount of overlap expected due to random chance is displayed in brackets below the actual observed overlap. The fold-enrichment over random expectation is displayed below each Venn diagram, as well as the cumulative probability of observing an overlap of equal or greater amount, as computed using the cumulative hypergeometric distribution function. p-values lower than 0.05 are indicated in red.

Tables S1-S14

Tables S1-S14 are available for download at http://www.g3journal.org/lookup/suppl/doi:10.1534/g3.111.000836/-/DC1.

Table S1 Homozygous deletion mutants with reduced fitness during fermentation. List of the 300 diploid homozygous deletion strains that exhibit a two-fold or greater decrease in barcode signal intensity in experimental over control sample in three or more time points.

Table S2 Homozygous deletion mutants with increased fitness during fermentation. List of the 303 diploid homozygous deletion strains that exhibit a two-fold or greater increase in barcode signal intensity in experimental over control sample in three or more time points.

Table S3 Homozygous deletion mutant pool fermentation fitness profiles. Complete dataset for all barcode signals from the homozygous deletion mutants during the fermentation time course.

Table S4 Heterozygous deletion mutants with reduced fitness during fermentation. List of the 481 diploid heterozygous deletion strains that exhibit a two-fold or greater decrease in barcode signal intensity in experimental over control sample in three or more time points.

Table S5 Heterozygous deletion mutants with increased fitness during fermentation. List of the 466 diploid heterozygous deletion strains that exhibit a two-fold or greater increase in barcode signal intensity in experimental over control sample in three or more time points.

Table S6 Heterozygous deletion mutant pool fermentation fitness profiles. Complete dataset for all barcode signals from the heterozygous deletion mutants during the fermentation time course.

Table S7 Overlap between homozygous and heterozygous deletion mutants with reduced fitness during fermentation. The 38 genes with reduced fitness in both the homozygous and heterozygous fermentation datasets are listed.

Table S8 Overlap between homozygous and heterozygous deletion mutants with increased fitness during fermentation. The 48 genes with increased fitness in both the homozygous and heterozygous fermentation datasets are listed.

Table S9 Homozygous deletion mutants with reduced fitness during fermentation in three or more sequential time points. List of the 180 diploid homozygous deletion strains that exhibit a two-fold or greater decrease in barcode signal intensity in experimental over control sample in three or more sequential time points.

Table S10 Homozygous deletion mutants with increased fitness during fermentation in three or more sequential time points. List of the 162 diploid homozygous deletion strains that exhibit a two-fold or greater increase in barcode signal intensity in experimental over control sample in three or more sequential time points.

Table S11 Heterozygous deletion mutants with reduced fitness during fermentation in three or more sequential time points. List of the 210 diploid heterozygous deletion strains that exhibit a two-fold or greater decrease in barcode signal intensity in experimental over control sample in three or more sequential time points.

Table S12 Heterozygous deletion mutants with increased fitness during fermentation in three or more sequential time points. List of the 214 diploid heterozygous deletion strains that exhibit a two-fold or greater increase in barcode signal intensity in experimental over control sample in three or more sequential time points.

Table S13 Overlap between homozygous and heterozygous deletion mutants with reduced fitness during fermentation in three or more sequential time points. The 20 genes with reduced fitness in both the homozygous and heterozygous sequential datasets are listed.

Table S14 Overlap between homozygous and heterozygous deletion mutants with increased fitness during fermentation in three or more sequential time points. The 18 genes with increased fitness in both the homozygous and heterozygous sequential datasets are listed.

Reference

DELNERI, D., D. C. HOYLE, K. GKARGKAS, E. J. CROSS, B. RASH *et al.*, 2008 Identification and characterization of high-flux-control genes of yeast through competition analyses in continuous cultures. Nat Genet **40:** 113-117.
