# Supplementary material for: Genome-wide Fitness Profiles Reveal a Requirement for Autophagy During Yeast Fermentation
Source: G3 (Bethesda). 2011 Oct 1;1(5):353–67. doi: 10.1534/g3.111.000836 (PMC3276155; doi:10.1534/g3.111.000836)
Supplement: Supporting Information [file supp_1.5.353_FigureS1.pdf]

A

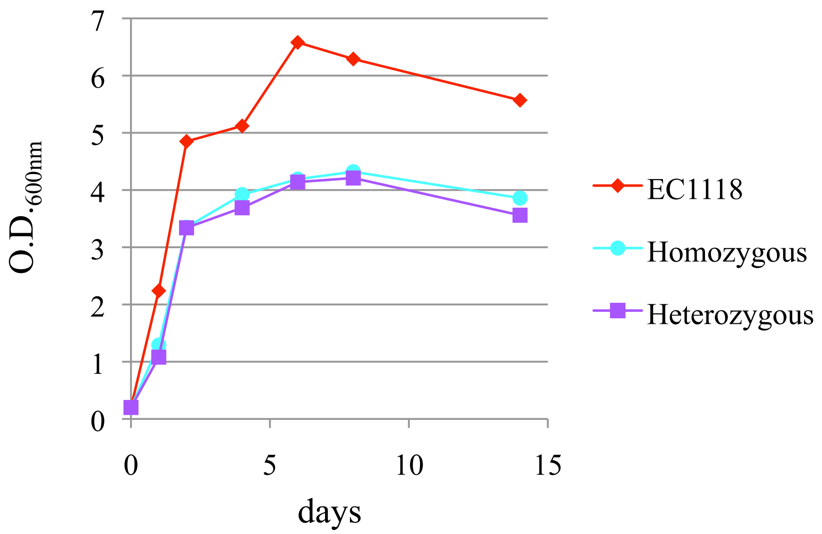

B

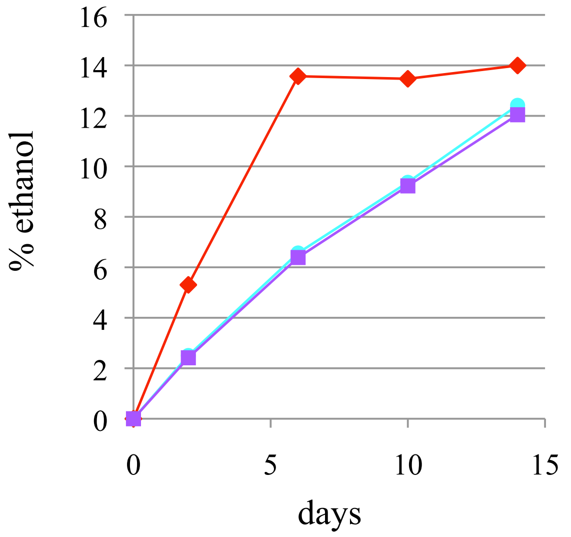

Figure S1 Fermentation of the homozygous and heterozygous yeast deletion pools (S288C) and the EC1118 wine yeast strain in synthetic grape juice. Samples extracted from these fermentations were used for genome-wide fitness profiling. (A) Cell growth curve measured by $O.D._{600nm}$, (B) ethanol production.
