# Supplementary material for: Genome-wide Fitness Profiles Reveal a Requirement for Autophagy During Yeast Fermentation
Source: G3 (Bethesda). 2011 Oct 1;1(5):353–67. doi: 10.1534/g3.111.000836 (PMC3276155; doi:10.1534/g3.111.000836)
Supplement: Supporting Information [file supp_1.5.353_FigureS2.pdf]

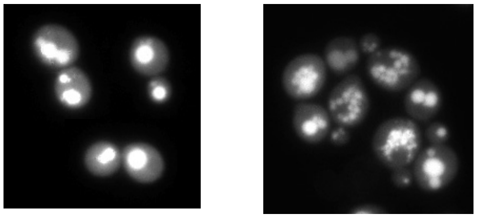

Log phase

Day1 fermentation

Figure S2 Vacuoles fragment during fermentation. Diploid wild type yeast cells were imaged during log phase growth and after one day of fermentation in synthetic grape juice. Vacuoles were stained with the vacuole-specific dye CMAC (7-amino-4-chloromethylcoumarin) and imaged using a fluorescence microscope.
