# Supplementary material for: Genome-wide Fitness Profiles Reveal a Requirement for Autophagy During Yeast Fermentation
Source: G3 (Bethesda). 2011 Oct 1;1(5):353–67. doi: 10.1534/g3.111.000836 (PMC3276155; doi:10.1534/g3.111.000836)
Supplement: Supporting Information [file supp_1.5.353_FigureS3.pdf]

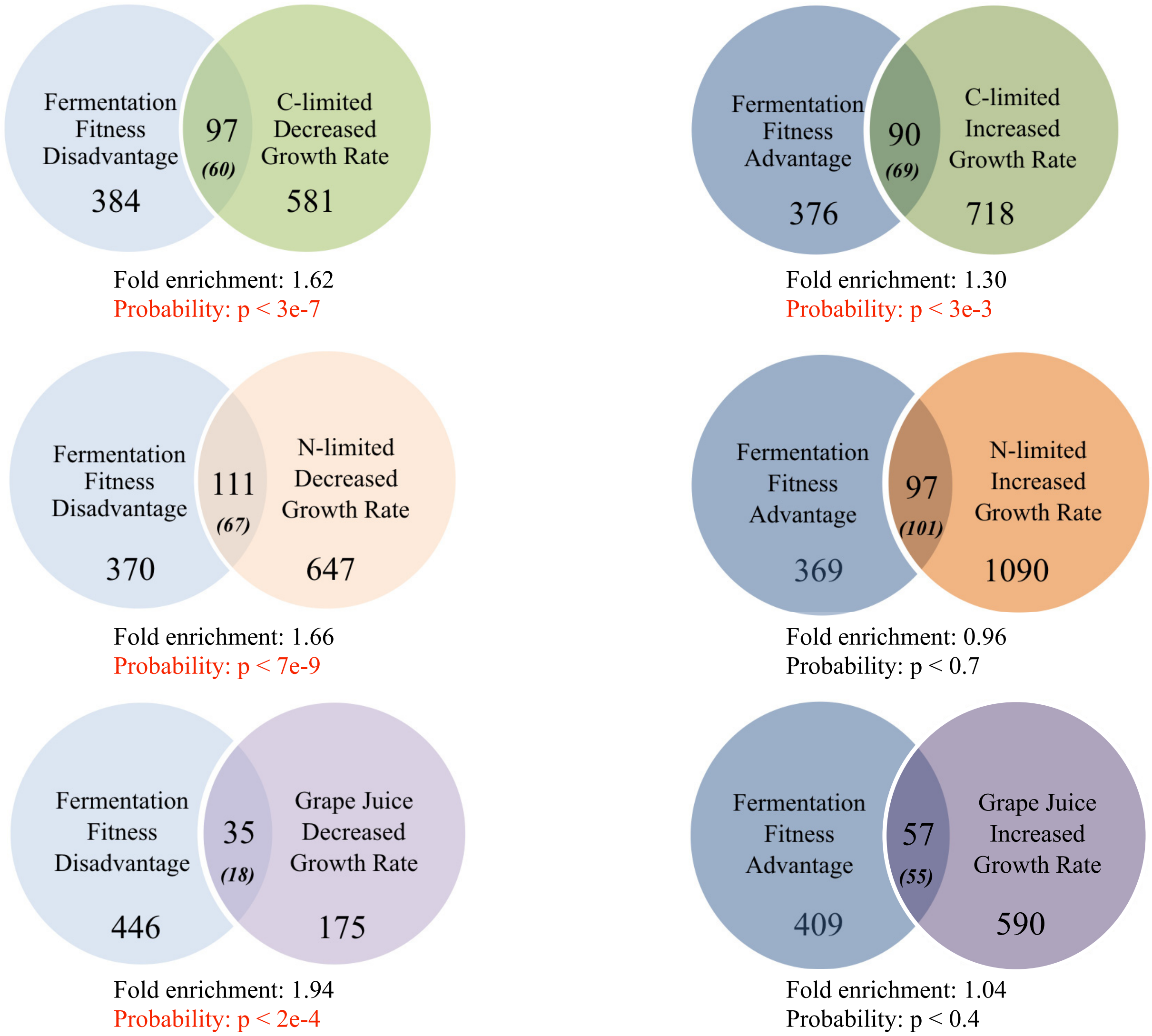

Figure S3 Heterozygous deletion mutants with decreased fitness during fermentation are enriched for mutants with reduced growth rates in nutrient limiting conditions. The heterozygous deletion mutants with reduced or increased fitness during fermentation were compared to mutants with decreased or increased growth rate in nitrogen-limiting (N-limited), carbon-limiting (C-limited) and grape juice media (DELNERI *et al.* 2008). The unique genes in each dataset as well as the overlap are displayed using a Venn diagram. The amount of overlap expected due to random chance is displayed in brackets below the actual observed overlap. The fold-enrichment over random expectation is displayed below each Venn diagram, as well as the cumulative probability of observing an overlap of equal or greater amount, as computed using the cumulative hypergeometric distribution function. p-values lower than 0.05 are indicated in red.
